# Anti-apoptotic proteins in the autophagic world: an update on functions of XIAP, Survivin, and BRUCE

**DOI:** 10.1186/s12929-020-0627-5

**Published:** 2020-02-05

**Authors:** Chun Hei Antonio Cheung, Yung-Chieh Chang, Tzu-Yu Lin, Siao Muk Cheng, Euphemia Leung

**Affiliations:** 1 Institute of Basic Medical Sciences, College of Medicine, National Cheng Kung University, No. 1 University Road, Tainan, Taiwan; 2 Department of Pharmacology, College of Medicine, National Cheng Kung University, Tainan, Taiwan; 3 National Institute of Cancer Research, National Health Research Institutes (NHRI), Tainan, Taiwan; 4 Auckland Cancer Society Research Centre, University of Auckland, 85 Park Rd, Grafton, Auckland, 1023 New Zealand; 5 Maurice Wilkins Centre for Molecular Biodiscovery, University of Auckland, Symonds Street, Auckland, 1010 New Zealand

**Keywords:** Autophagy, Apoptosis, BRUCE, IAP, Survivin, Smac, XIAP

## Abstract

X-linked inhibitor of apoptosis protein (XIAP), survivin, and BRUCE are members of the inhibitor-of-apoptosis protein (IAP) family known for their inhibitory effects on caspase activity and dysregulation of these molecules has widely been shown to cause embryonic defects and to promote tumorigenesis in human. Besides the anti-apoptotic functions, recent discoveries have revealed that XIAP, survivin, and BRUCE also exhibit regulatory functions for autophagy in cells. As the role of autophagy in human diseases has already been discussed extensively in different reviews; in this review, we will discuss the emerging autophagic role of XIAP, survivin, and BRUCE in cancer cells. We also provide an update on the anti-apoptotic functions and the roles in maintaining DNA integrity of these molecules. Second mitochondria-derived activator of caspases (Smac) is a pro-apoptotic protein and IAPs are the molecular targets of various Smac mimetics currently under clinical trials. Better understanding on the functions of XIAP, survivin, and BRUCE can enable us to predict possible side effects of these drugs and to design a more “patient-specific” clinical trial for Smac mimetics in the future.

## Introduction

Apoptosis is a cellular process highly regulated by different pro-apoptotic and anti-apoptotic proteins, like members of the inhibitor-of-apoptosis protein (IAP) family and the Bcl-2 family. Currently, there are eight IAP family members in human - cIAP1, cIAP2, ML-IAP/Livin, Ts-IAP/ILP-2, NIAP, XIAP, survivin, and BRUCE. Structurally, IAP family members are characterized by the presence of at least one Baculoviral IAP Repeat (BIR) domain (Table 1) and it has widely been demonstrated that the presence of the BIR domain is crucial for IAPs to inhibit the activity of different caspases through physical interactions. As IAP family members regulate a variety of cellular physiological processes [1–3] and dysregulations (i.e. mostly upregulation) of these molecules are known to promote tumorigenesis, tumor metastasis, and anti-cancer therapy resistance in human [4–9], it is important to understand the biology of different IAP family members and the mechanism underlying the dysregulation of these molecules in cancer cells. Although some of the IAP family members have already been known for more than two decades and several anti-cancer small-molecule Smac mimetics (i.e. a class of IAPs-targeting compounds) have been developed and reached clinical trials [10–12], scientists still not yet fully understand their molecular functions in cancer cells.

**Table 1:** Different IAP family members of *Homo sapiens*

Name	Location (chromosome locus)	Length of coding sequence (bp)	Molecular weight of protein (kDa)	Number of BIR domain	Number of RING domain
NAIP	5q13.2	4212	159.6	3 (BIR 1, 2, and 3)	–
cIAP1	11q22.2	1857	69.9	3 (BIR 1, 2, and 3)	1
cIAP2	11q22.2	1815	68.4	3 (BIR 1, 2, and 3)	1
XIAP	Xq25	1494	56.7	3 (BIR 1, 2, and 3)	1
Survivin	17q25.3	429	16.4	1 (BIR)	–
BRUCE	2p22.3	14,574	530.3	1 (BIR)	–
Livin	20q13.33	897	32.8	1 (BIR 3)	1
ILP-2	19q13.42	711	27.1	1 (BIR 3)	1

Autophagy is currently one of the hottest topics in cancer research. Despite intensive research has been conducted in the past decade to better understand the process of autophagy [13–23], the detailed regulatory mechanism and cellular effects are still not yet fully understood. Generally, autophagy is a dynamic catabolic process used for removing unnecessary or dysfunctional proteins and organelles in cells. Pathologically, dysregulation of autophagy promotes tumorigenesis and upregulation of autophagy has widely been shown to provide extra survival signals in both normal and cancer cells exposed to various internal and external stresses [14–22]. For example, hypoxia-induced autophagy process might contribute to the resistance to chemotherapeutic agent, cisplatin, in non-small cell lung cancer [19]. The process of apoptosis and autophagy was believed to be mutually exclusive; however, emerging evidence suggests that they are inter-connected and inter-regulated at the molecular level (e.g. through Bcl-2) in cells. In the following sections, we will discuss the lately discovered autophagic role of the well-known anti-apoptotic molecules, XIAP, survivin, and BRUCE.

### XIAP as a regulator of apoptosis and necroptosis

**Fig. 1:**
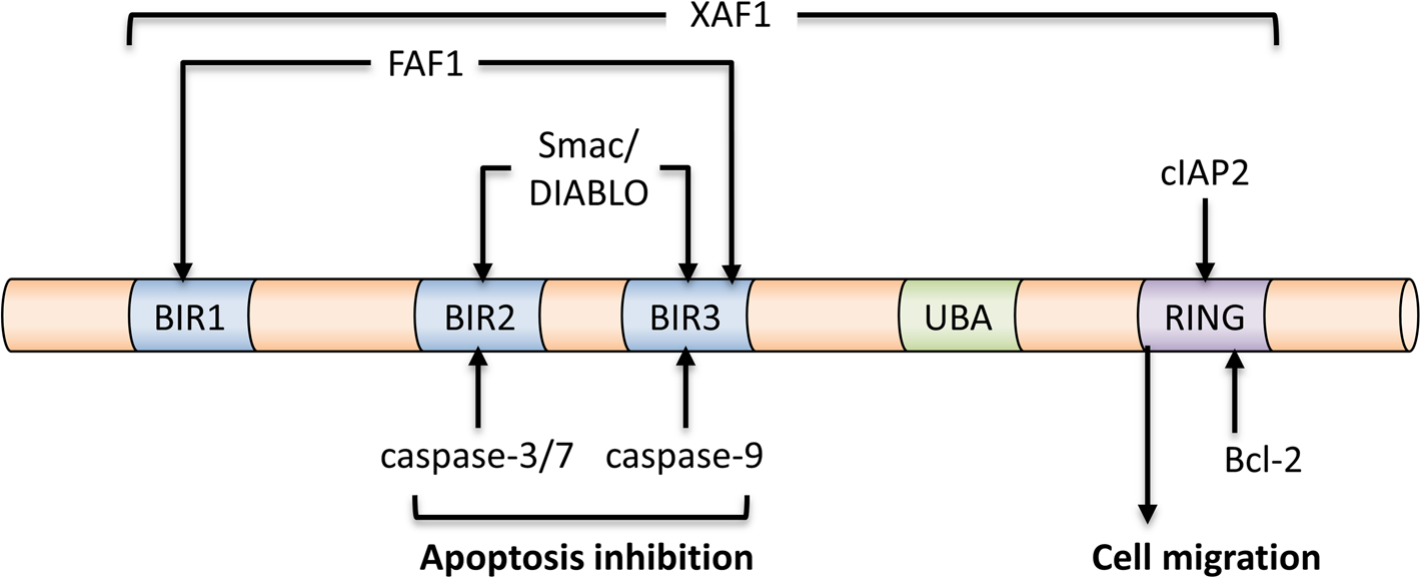
Identified binding partners of XIAP

XIAP, discovered in 1996, contains three BIR domains (BIR1, BIR2, and BIR3) and a single Really Interesting New Gene (RING) finger domain (Fig. 1). As an apoptosis inhibitor, the caspase-3 and -7 inhibiting activity has been localized to the BIR2 domain and the BIR3 domain of XIAP is responsible for the inhibition of caspase-9 [24, 25]. In contrast, the RING domain of XIAP exhibits E3 ubiquitin ligase activity and this activity is required for the XIAP-mediated cancer cell migration [26–28]. Besides interacting with caspase-9 and caspase-3, XIAP also directly or indirectly interacts with different IAPs and Smac [also known as direct inhibitor of apoptosis-binding protein with low pI (DIABLO)] [29–32]. The RING finger domain of XIAP is capable of interacting with the BIR2 and BIR3 domain of cIAP2 and this XIAP-cIAP2 complexation upregulates the protein stability of cIAP2 in glioblastoma cells [33]. On the other hand, formation of the survivin-XIAP complex prevents XIAP undergoing polyubiquitination and the subsequent proteasomal degradation, thereby stabilizing XIAP in cancer cells [29]. In contrast, Smac is a known pro-apoptotic molecule and formation of the Smac-XIAP complex prevents XIAP binding to different caspases and promotes cellular apoptosis [30–32]. A recent study by Caballero-Lopez et al. reveals that XIAP binds to the pro-apoptotic molecule, FAS-associated factor 1 (FAF1), leads to the polyubiquitination and degradation of this molecule, and consequently inhibits FAF1-mediated cell death in cancer cells [34]. However, the effects of the E3 ubiquitin ligase activity of XIAP seems not to be “pro-apoptotic molecule specific” as XIAP also stimulates ubiquitin proteasome system (UPS)-mediated degradation of the anti-apoptotic molecule, Bcl-2, to promote apoptosis upon the formation of an XIAP- apoptosis related protein in TGF-β signaling pathway (ARTS)-Bcl-2 ternary complex [35]. These findings are indeed interesting because they suggest that even though XIAP exhibits both anti-apoptotic and pro-apoptotic activities, the anti-apoptotic activity seems to be prominent as overexpression of XIAP has widely been demonstrated to promote cells survival and tumorigenesis, whereas, downregulation of this molecule promotes cancer cells death. Besides apoptosis, cells can also undergo a specific type of programmed self-destruction called necroptosis. Necroptosis is a form of programmed cell death mediated by receptor-interacting kinase 1 (RIPK1), RIPK3, and mixed lineage kinase domain-like protein (MLKL). It is now clear that that XIAP also plays an important role in regulating necroptosis in innate immune cells [36–38]. For example, loss of XIAP has been shown to promote the switch from tumor necrosis factor-α (TNFα; at high concentrations)-induced apoptosis to RIPK3-dependent necroptosis in mouse neutrophils [37].

### XIAP as a controversial autophagy modulator

**Fig. 2:**
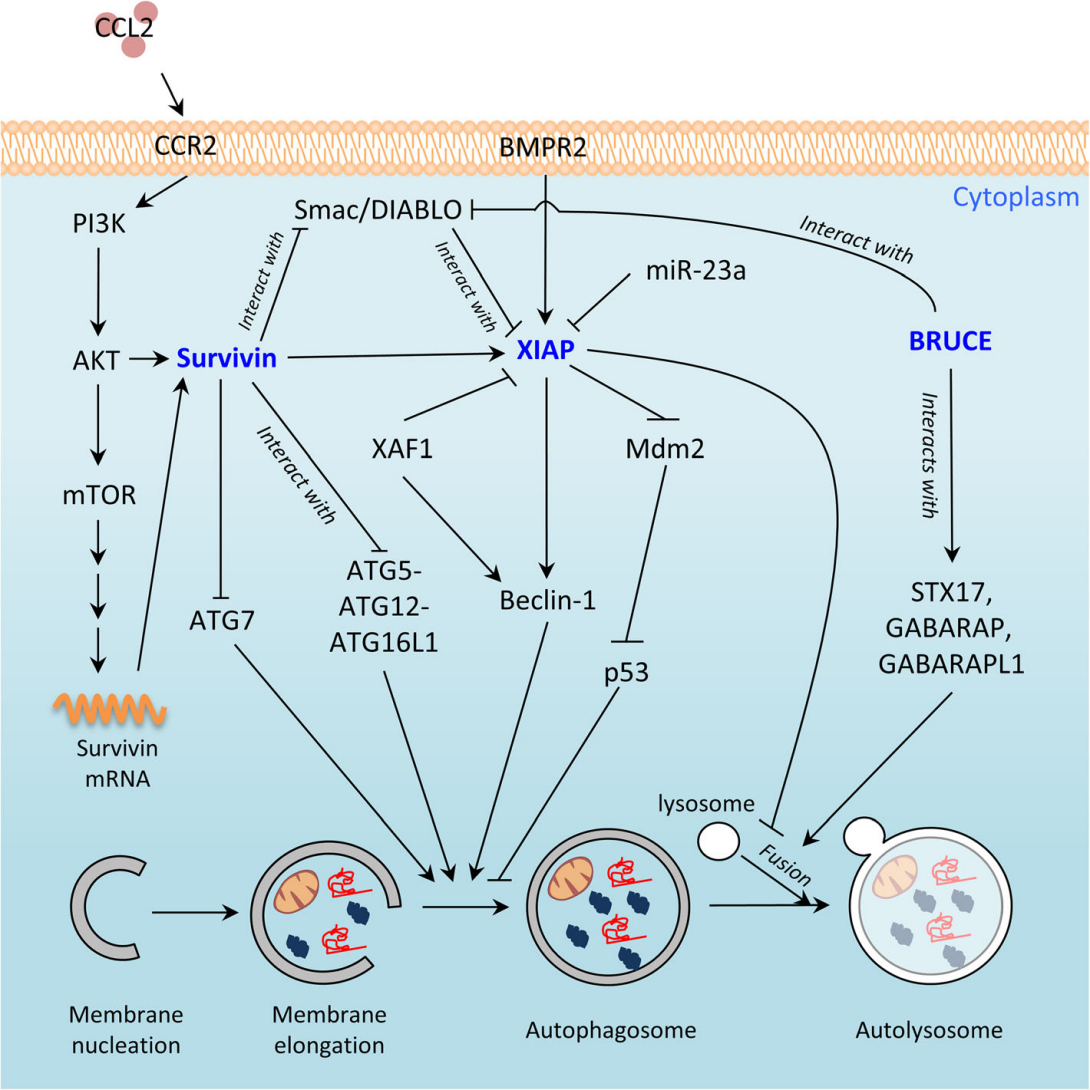
Schematic diagram showing the interactions between XIAP, survivin, BRUCE, and other molecules in the regulation of cellular autophagy

Despite XIAP was originally discovered as an inhibitor of caspases and apoptosis, a number of studies suggest that XIAP is an autophagy modulator. An inverse correlation in the expression between XIAP and a known autophagy-related molecule, microtubule-associated protein light chain 3 (LC3), in hepatocellular carcinoma tissue specimens has been reported in the past [39]. The most direct evidence supporting its role as an autophagy negative-regulator came from a study by Huang et al. In this study, XIAP was shown to be capable of inhibiting autophagy via a XIAP-Mouse double minute 2 homolog (Mdm2)-p53 signaling pathway in the wild-type p53 (p53^WT^)-expressing HCT116 cancer cells, but not in the p53^−/−^ HCT116 cancer cells [40]. Bone morphogenetic protein receptor 2 (BMPR2) is a growth factor receptor and downregulation of BMPR2 by siRNA was demonstrated to induce autophagy in chondrosarcoma cells, again, via the XIAP-Mdm2-p53 signaling pathway [41]. Recent studies further reveal that direct or indirect inhibitions/downregulations of XIAP can promote the induction of cellular autophagy. For example, the microRNA miR-23a was found to be a negative regulator of XIAP (i.e. downregulates the expression) and overexpression of miR-23a was shown to upregulate the endogenous autophagic levels of breast cancer cells in a XIAP-dependent manner (Fig. 2) [42]. Embelin (2,5-dihydroxy-3-undecyl-2,5-cyclohexadiene-1,4-dione) is a natural compound isolated from *Embelia ribes* [43]. Lee et al. showed that inhibiting XIAP by embelin induced autophagy in the human oral Ca9–22 squamous carcinoma cells in vitro [44]. Furthermore, it has been demonstrated that adenovirus vector-mediated XIAP-associated factor 1 (XAF1) expression induces autophagy and autophagic cell death via Beclin-1 upregulation in gastric cancer cells [45]. Of note, XAF1 is a known XIAP molecular antagonist that negatively modulates the caspase inhibitory function of XIAP through physical interactions and the subsequent redistribution of XIAP from the cytoplasm to the nucleus [46].

XIAP has also been suggested as an autophagy upregulator. Even though targeting IAPs including XIAP, cIAP1, and cIAP2 by a Smac mimetic, APG-1387, was shown to induce autophagy and cell death in human ovarian cancer cells [47]; contrary, addition of a different Smac mimetic, LCL161 (a drug known to target cIAP1, cIAP2, and XIAP), at high dose was shown to inhibit the fusion between autophagosome and lysosome in mouse embryonic cells (MEFs) [48]. Downregulations of cIAP2 and XIAP by siRNA were demonstrated to induce similar cellular phenotypes in MEFs [48], further suggesting that XIAP can act as an autophagy suppressor, despite the detailed molecular mechanism remains to be determined. Noticeably, XIAP and cIAP1 have also been suggested to positively-regulate the expression of Beclin 1, which is a protein crucial for the biogenesis of autophagosome during canonical autophagy, via an nuclear factor-κB (NFκB)-signaling pathway [49]. Thus, XIAP seems to exhibit differential autophagic roles in different cells under different circumstances.

### Survivin as an apoptosis inhibitor and a mitosis positive regulator

Survivin, discovered in 1997, is the smallest member of the IAP family proteins and it contains only a single BIR domain. Similar to other IAP family members, survivin is believed or has been demonstrated to be an apoptosis negative-regulator [50]. For example, Chandele et al. showed that survivin inhibited caspase-9 activity and promoted staurosporine-resistance in human SK-N-MC neuroblastoma cells [51]. A purified recombinant human survivin protein expressed in *E. coli* was shown capable of binding to caspase-3 and caspase-7 in solution [52]. Furthermore, activation of caspase-3 and induction of apoptosis were widely observed in cancer cells with survivin downregulations or inhibitions [53–59]. As aforementioned, Smac is a negative-regulator of XIAP and it promotes caspase activation and apoptosis through formation of the XIAP-Smac protein complex. As an anti-apoptotic molecule, survivin binds to Smac and consequently prevents this molecule from binding onto XIAP, resulting in the inhibition of caspase-9 and caspase-3 [60–62]. In addition, it has been shown that survivin negatively modulates the activation of caspase-independent apoptosis through regulation of the nuclear translocation of apoptosis-inducing factor (AIF) [63].

Unlike other IAP family members, survivin also plays an important role in mitosis. At the molecular level, survivin forms the chromosomal passenger complex (CPC) with inner centromere protein (INCENP), borealin (also known as Dasra), and Aurora B kinase and proper formation (and localization) of the CPC during M phase of the cell cycle are both crucial for the completion of mitosis [64, 65]. Interestingly, a recent study revealed that the survivin homodimer interacts with myosin II to regulate cytokinesis [66]. Therefore, survivin is widely accepted as a multi-functions protein, which is capable of inhibiting caspase-dependent and -independent apoptosis through both direct and indirect modulations and promoting mitosis through formation of the CPC in cancer cells.

### Survivin negatively modulates autophagy

Emerging evidence indicates that survivin is a negative regulator of autophagy. For example, the small molecule survivin suppressant, YM155, was shown to induce the death of salivary adenoid cystic carcinoma, breast cancer, and the Bcl-xL silenced glioma cells in an autophagy-dependent manner [67–69]. Despite autophagy upregulation is known to promote homologous recombination and DNA repair in cells under genotoxic stress [70, 71], Cheng et al. demonstrated YM155 also induces autophagy-dependent DNA damage in breast cancer cells regardless to the expression of p53 and caspase-3 [68]. Moreover, delivery of a survivin promoter-driven antisense survivin-expressing plasmid DNA was shown to induce apoptosis and autophagy in A549, MDA-MB-231, and PANC-1 cancer cells in vitro [58]. Conversely, survivin overexpression inhibits autophagy. For example, chemokine (C-C motif) ligand 2 (CCL2, also known as MCP1) was found to protect human PC3 prostate cancer cells from undergoing autophagic death via PI3K/AKT-dependent survivin upregulations (Fig. 2) [72].

Mechanistically, survivin suppresses autophagy possibly through interference with the development of autophagosome in cells [73]. It has been demonstrated autophagy related protein 5 (ATG5) interacts with survivin to displace Aurora B kinase from survivin in the nucleus in MDA-MB-231 breast cancer cells treated with DNA-damaging agents [74]. Interestingly, we recently discovered that survivin inhibits the conjugation between autophagy related protein 12 (ATG12) and ATG5 (i.e. the formation of ATG12-ATG5 conjugate) through physical interactions with both ATG12 (i.e. ATG12-survivin complexation) and ATG5 (i.e. ATG5-survivin complexation) [75]. We also found that survivin binds to ATG12-ATG5 conjugate (i.e. ATG12-ATG5-survivin complexation) and inhibits the formation of ATG12-ATG5-ATG16L1 in human cancer and mouse embryonic fibroblast cells (Fig. 2) [75]. Besides inhibiting the conjugation and complexation between ATG12, ATG5, and ATG16L1, survivin also negatively modulates the protein stability of autophagy related protein 7 (ATG7; a protein that facilitates LC3 lipidation) in part through an heat shock protein 27 (Hsp27) dependent mechanism [75]. Given that successful formation of the ATG12-ATG5-ATG16L1 protein complex is crucial for the elongation of autophagophore during canonical autophagy, inhibiting the formation of this protein complex shall block the autophagic flux in cells (Fig. 2).

It is worth noting that the translation of survivin mRNA transcripts is positively regulated by the AKT/mTOR signaling pathway and targeting this signaling pathway by small molecule inhibitor, rapamycin, has been shown to induce autophagy in cells [76–80]. Furthermore, as mentioned, XIAP inhibits autophagy via an XIAP-Mdm2-p53 signaling pathway in p53^WT^-expressing cancer cells. Thus, survivin may inhibit autophagy in part through interference with the XIAP-Mdm2-p53 pathway in p53^WT^-expressing cells. Collectively, even though the detailed mechanistic role of XIAP and survivin on autophagy regulation remains to be fully elucidated, especially in p53^−/−^ and p53^mutant^ expressing cells; however, it is clear that XIAP and survivin are not solely an apoptosis inhibitor but a dual/multi-functions protein, which participates in both apoptosis, mitosis, and autophagy regulations in cells.

### BRUCE mediates homologous recombination and autophagosome-lysosome fusion

BIR repeat containing ubiquitin-conjugating enzyme (BRUCE, also known as Apollon) was discovered in 1998 as a member of IAPs family [81]. Structurally, it contains a single BIR domain and a single Ubiquitin-conjugating enzymes (UBC) domain (i.e. exhibits E2/E3 ubiquitin ligase activity) [82, 83]. Mechanistically, BRUCE inhibits apoptosis through physical interactions with DIABLO/Smac and caspase-9 and promotes their degradation through protein ubiquitination [84, 85]. Like survivin, BRUCE was also found to exhibit caspase inhibitory unrelated functions in cells. Breast cancer susceptibility gene C terminus-repeat inhibitor of human telomerase repeat transcriptase expression 1 (BRIT1) is an early double damage response factor. During DNA damage, BRIT1 is recruited to the phosphated-H2AX (γ-H2AX) attached DNA double-strand breaks and subsequently to facilitate DNA repair. Downregulation of BRUCE was shown to inhibit the ataxia-telangiectasia mutated and RAD3-related (ATR)-signaling pathway and to impair BRIT1 deubiquitinationin in U2OS cells. As demonstrated by Ge et al., the presence of BRUCE is crucial during DNA replication and the DNA double-strand breaks repair [86, 87]. Besides acting as an apoptosis inhibitor, a study by Kikuchi et al. showed that BRUCE also regulates mitosis through modulating the ubiquitylation and protein stability of cyclin A [88].

Recent evidence suggests that BRUCE may play a role in the formation of autolysosome (autophagosome-lysosome fusion). As described in the above sections, autophagosome and autolysosome formations are medicated by both sequential activations and complex formations between different ATG family proteins. Among these ATG family proteins, Autophagy related protein 8 (ATG8) family proteins such as LC3, GABA type A receptor-associated protein (GABARAP), and GABARAP-LIKE 1 (GABARAPL1/GEC1) govern the fusion between autophagosome and lysosome (i.e. formation of autolysosome) [89]. An interesting study by Ebner et al. revealed that BRUCE physically interacts with syntaxin 17 (STX17), GABARAP, and GABARAPL1, to promote autophagosome-lysosome fusion in mammalian cells independent of its catalytic function (Fig. 2) [90].

### IAPs as “pro-survival autophagy” guardians?

**Fig. 3:**
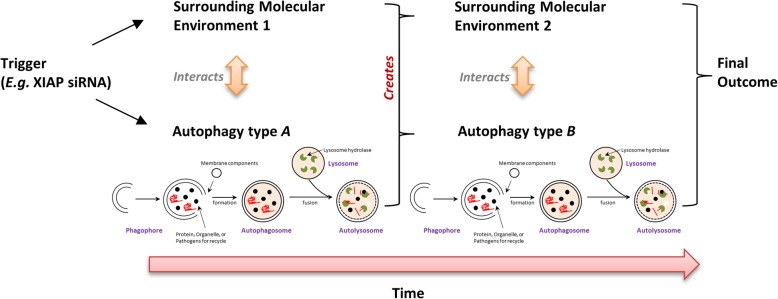
The dynamic autophagic environments model

Upregulation of autophagy has been shown to promote the survival of cancer and cancer-related cells treated with a variety of therapeutics including tamoxifen, paclitaxel, epirubicin, and azacytidine [14–18]. Autophagy is also known to assist homologous recombination, which is a type of DNA repair mechanisms, in cells treated with DNA damaging agents and UV radiation [91–93]. As aforementioned, overexpression of IAPs has widely been demonstrated to inhibit chemotherapeutic/targeted therapeutic drugs induced apoptosis in cancer cells. Therefore, it is unclear on the reason of having IAPs such as XIAP and survivin as autophagy suppressors, given that upregulation of autophagy and IAPs should both promote the survival of cancer cells, especially under cellular stressful conditions. Perhaps the main function of XIAP, survivin, and BRUCE on autophagy is not to largely promote or suppress this process, but to fine tune and to maintain the level of autophagy within certain “pro-survival” ranges. Despite upregulation of autophagy is widely believed to promote DNA repair (like homologous recombination), a few studies showed that excessive activation of autophagy causes DNA damage in cells. For example, it has been demonstrated that targeting cathepsin S (CTSS) induces autophagy, leading to the autophagy-dependent reactive oxygen species (ROS) production and DNA damage in OEC-M1 cells [94]. A study by Chen et al. showed that upregulation of autophagy decreases the intracellular pool of deoxyribonucleotide triphosphate (dNTP) in Huh-7 cells treated with Earle’s balanced salt solution (EBSS) or rapamycin [95]. We also demonstrated that downregulating survivin by YM155 and siRNA induces autophagy-dependent DNA damage and cell death in human cancer cells [68, 75]. So clearly, excessive autophagy (passing certain thresholds) can cause genomic instability, and by altering the expression, post-translational modification, and subcellular-localization of XIAP, survivin, and BRUCE, cells can precisely regulate the autophagy level to maintain their survival under stressful conditions. However, if XIAP, survivin, and BRUCE are three of the “guardians” of the “pro-survival autophagy” (via fine tuning the autophagic level of cells), then why contradicting results were frequently reported regarding to the role of the “induced autophagy” (i.e. autophagy-promoted survival cell or autophagy-induced cell death) in cells treated with agents targeting XIAP and BRUCE? As most IAPs can directly or indirectly interact with multiple molecules, which regulate different molecular and cellular processes like DNA repair and mitosis, the observed “resulting autophagic effects” probably were not solely caused by the direct protein-protein interaction effects of these IAPs on different autophagy core molecules, but were results of the dynamic crosstalk between different IAPs-involved molecular and cellular processes (Fig. 3). As the “weight” of each of these processes varies under different cellular environments or treatments, the autophagic outcome can be completely different. Therefore, besides understanding the direct effects of XIAP, survivin, and BRUCE on various autophagic/apoptotic/mitotic components, it is also important to understand the dynamic interactions between the autophagic process and the surrounding molecular environments within the cell.

## Conclusion and future directions

**Fig. 4:**
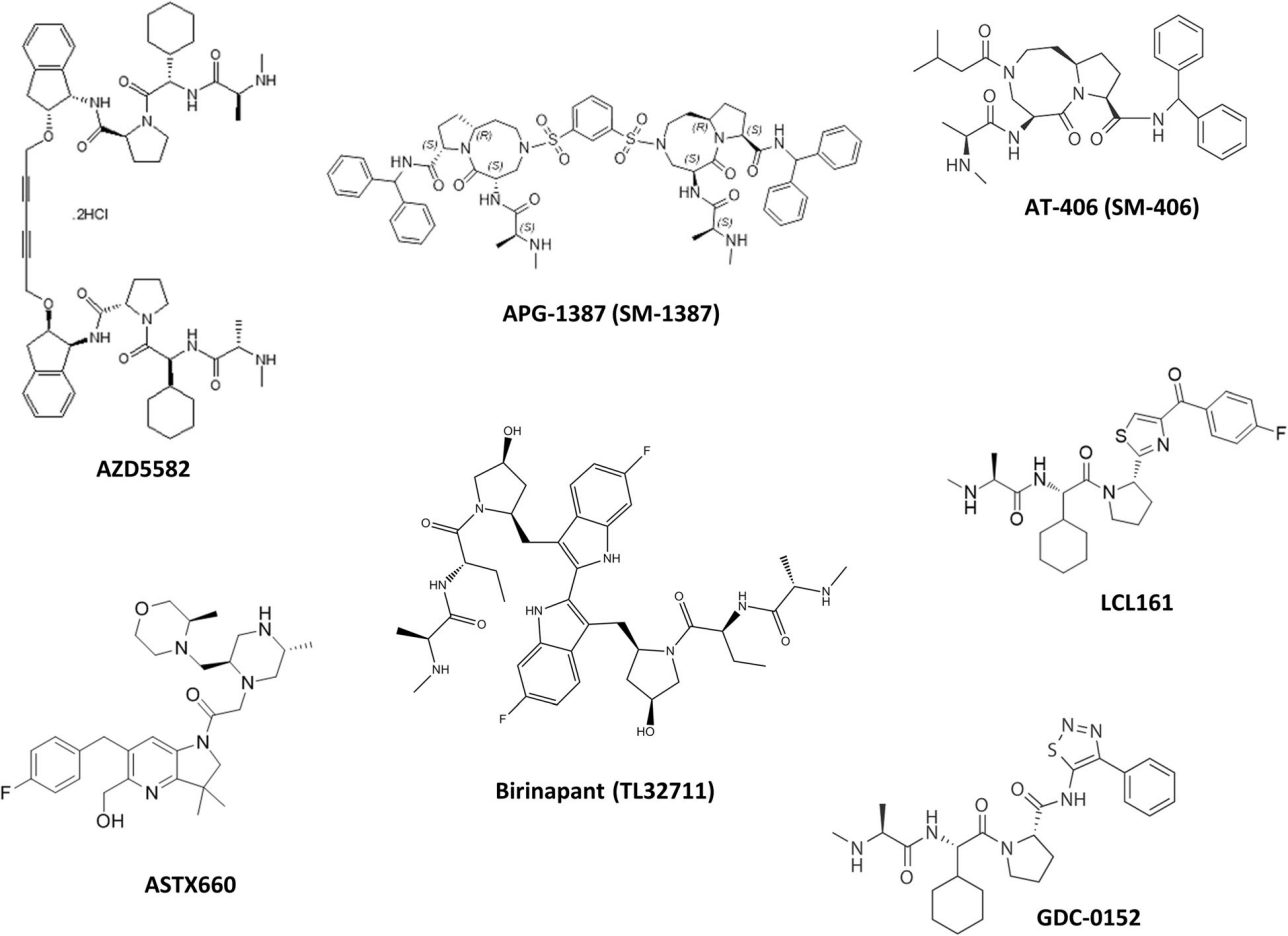
The chemical structure of different Smac mimetics developed for cancer treatments

IAP family members are traditionally classified as caspase inhibitors with negative-modulating effects on cellular apoptosis. However, emerging evidence suggest that these molecules can also regulate cellular autophagy. It is not surprising that the anti-apoptotic molecules XIAP, survivin, and BRUCE are capable of modulating autophagy, given that the well-studied anti-apoptotic molecule, Bcl-2, is also known to be an apoptosis-autophagy dual modulator (i.e. inhibits Beclin 1-dependent autophagy) in cells [96]. As mitosis, apoptosis, and autophagy are inter-connected, XIAP, survivin, BRUCE, and Bcl-2 may act as bridging molecules that control the dynamics and the balance between these cellular processes. For example, cancer cells can upregulate autophagy to produce the “minimal” energy needed for their survival under serum deprivation. In addition, cancer cells can also temporarily halt mitosis, probably to spare energy, under serum deprivation. In fact, we found in a previous study that serum deprivation decreases the complexation between survivin and ATG12/ATG5 (possibly to upregulate autophagy), but not caspase-3 (concurrently maintains apoptosis inhibition), in human cancer cells [75]. However, it is still unclear on how cancer cells regulate the expression and protein-protein interaction (i.e. binding-target switch) of these IAPs to inter-regulate apoptosis, mitosis, and autophagy under different circumstances (like under hypoxia and nutrient deprivation). Thus, further investigations are needed to understand the differential regulations of these IAPs at the molecular level in cancer and non-cancerous cells. As various Smac mimetics (IAP antagonists) are currently in different phases of clinical trial and pre-clinical development (Table 2) (Fig. 4) [12, 97–104], better understanding on the functions of IAPs (e.g. XIAP, survivin, and BRUCE) can enable us to predict possible side effects of the drugs and to design a more “patient-specific” clinical trial for Smac mimetics in the future.

**Table 2:** Status of different Smac mimetics

Name	ClinicalTrials.gov Identifier	Phase	Condition or disease (in patients)
AZD5582	–	Pre-clinical	–
APG-1387 (SM-1387)	NCT03386526	Phase I	Advanced Solid Tumors or Hematologic Malignancies
	NCT03585322	Phase I	Chronic Hepatitis B
ASTX660	NCT04155580	Phase I	Relapsed/Refractory Acute Myeloid Leukemia
	NCT02503423	Phase I/II	Advanced Solid Tumors and Lymphomas
Birinapant (TL32711)	NCT02587962	Phase I/II	Solid Tumors
	NCT00993239	Phase I (Completed)	Refractory Solid Tumors or Lymphoma
	NCT01188499	Phase I (Completed)	Advanced or Metastatic Solid Tumors
	NCT01940172	Phase I (Completed)	Relapsed Ovarian Cancer
	NCT01573780	Phase I (Terminated – safety unrelated issue)	Advanced Solid Tumors
	NCT01681368	Phase II (Terminated – lack of a clinical benefit)	Advanced Ovarian, Fallopian Tube, and Peritoneal Cancer
Debio 1143 (AT-406, SM-406)	NCT04122625	Phase I	Solid Tumor
	NCT03270176	Phase I	Advanced or Metastatic Non-Small Cell Lung Cancer (NSCLC) After Platinum-Based Therapy
	NCT03871959	Phase I	Pancreatic and Colorectal Advanced/Metastatic Adenocarcinoma
	NCT02022098	Phase I/II	Squamous Cell Carcinoma of the Head and Neck
	NCT01078649	Phase I (Completed)	Advanced Solid Tumors and Lymphomas
GDC-0152	NCT00977067	Phase I (Terminated – safety unrelated issue)	Locally Advanced or Metastatic Malignancies
LCL161	NCT02649673	Phase I	Relapsed/Refractory Small Cell Lung Cancer (SCLC) and Select Gynecologic Malignancies
	NCT03111992	Phase I	Multiple Myeloma
	NCT01968915	Phase I (Completed)	Advanced Solid Tumors
	NCT02098161	Phase II	Primary Myelofibrosis, Post-Polycythemia Vera Myelofibrosis, or Post-Essential Thrombocytosis Myelofibrosis
	NCT01955434	Phase II (Completed)	Relapsed or Refractory Multiple Myeloma
WX20120108	–	Pre-clinical	–

## Data Availability

Not applicable.

## Abbreviations

ATG: Autophagy related gene/protein
BIR: Baculovirus inhibitor of apoptosis protein repeat
BRIT1: Breast cancer susceptibility gene C terminus-repeat inhibitor of human telomerase repeat transcriptase expression 1
BRUCE: BIR repeat-containing ubiquitin-conjugating enzyme
cIAP1: Cellular inhibitor of apoptosis protein 1
cIAP2: Cellular inhibitor of apoptosis protein 2
DIABLO: Direct inhibitor of apoptosis-binding protein with low pI
GABARAP: GABA type A receptor-associated protein
IAP: Inhibitor-of-apoptosis protein
LC3: Microtubule-associated protein light chain 3
ML-IAP: Melanoma inhibitor of apoptosis protein
NIAP: Neuronal apoptosis inhibitory protein
Smac: Second mitochondrial activator of caspases
Ts-IAP: Testis-specific inhibitor of apoptosis protein
XIAP: X-linked inhibitor of apoptosis protein
